# Advancements to the Multi-System Model of Resilience: updates from empirical evidence

**DOI:** 10.1016/j.heliyon.2020.e04831

**Published:** 2020-09-14

**Authors:** Jenny J.W. Liu, Maureen Reed, Kenneth P. Fung

**Affiliations:** a Department of Psychiatry, Centre for Mental Health, University Health Network, Canada; b Department of Psychology, Ryerson University, Toronto, ON, Canada; c Department of Psychiatry, University of Toronto, Toronto, ON, Canada

**Keywords:** Psychology, Resilience, Adversity, Multi-system resilience, Psychological resilience, Community resilience, Resilience measurement, Resilience capacity

## Abstract

In this paper, we discuss further advancements to the Multi-System Model of Resilience through examining empirical factor structures of the Multi-System Model of Resilience Inventory along with other measures of resilience. Evidence from multiple sampled populations provided support for the three-systems organization of the model and highlight its similarities and differences in relation to other measures of resilience. The MSMR conceptualizes resilience as a capacity that enables functioning across a continuum from vulnerability to resilience, whereby internal and external resources interface with dynamic coping processes in response to varying needs and goals. Meaningful applications of this model and future steps in model and measurement developments are discussed.

## 1 Introduction

Researchers have undertaken various ways of operationalizing resilience, including as the capacity to adapt to challenges/stressors (Masten, 1994; Southwick et al., 2014), the trajectory of recovery following challenges (Bonanno, 2004; Zemba et al., 2019; Southwick et al., 2014), and foundational features that support access to resources in the community (Linkov and Trump, 2019; Panter-Brick, 2014). Resilience can encompass the dynamic process in response to challenges or adversity; the positive outcome of coping; and the potential capacity to mount adaptive responses to obtain desired outcomes. Various attributes of resilience and the inter-relationship among them may be studied, including: internal and external risks and protective factors (e.g., personality, family, community); exposure to stress and adversity (e.g., traumatic events, natural disasters); attributes that characterize resilience as a process (e.g., self-efficacy and determination); and outcomes of coping (e.g., post-traumatic growth) (Garcia-Dia et al., 2013; Linkov et al., 2020; Linkov and Trump, 2019).

Research on resilience have focused on the response to the occurrence of a particular stressor, challenge, or trauma. However, an individual is not limited to a single exposure to a traumatic event in his or her lifetime, nor does exposure to the same event warrant similar outcomes among different individuals. The assessment and understanding of resilience in one situation may not be applicable to a different context (Liu et al., 2017). The lack of consensus in understanding resilience through a comprehensive framework and the heterogeneity of approaches to measuring resilience compromise the generalizability of findings. Resilience is affected by many interacting factors, such as existing health status, coping strategies used, and accessibility to support services (Masten, 2014; Masten et al., 2010; Prince-Embury and Saklofske, 2012). Rather than only focusing on risk and vulnerabilities at the individual level, considerations should also be given to the socio-structural determinants, including access to and availability of resources that promote resilience (Liu et al., 2017; Panter-Brick, 2014).

### 1.1 The Multi-System Model of Resilience

**Figure 1:**
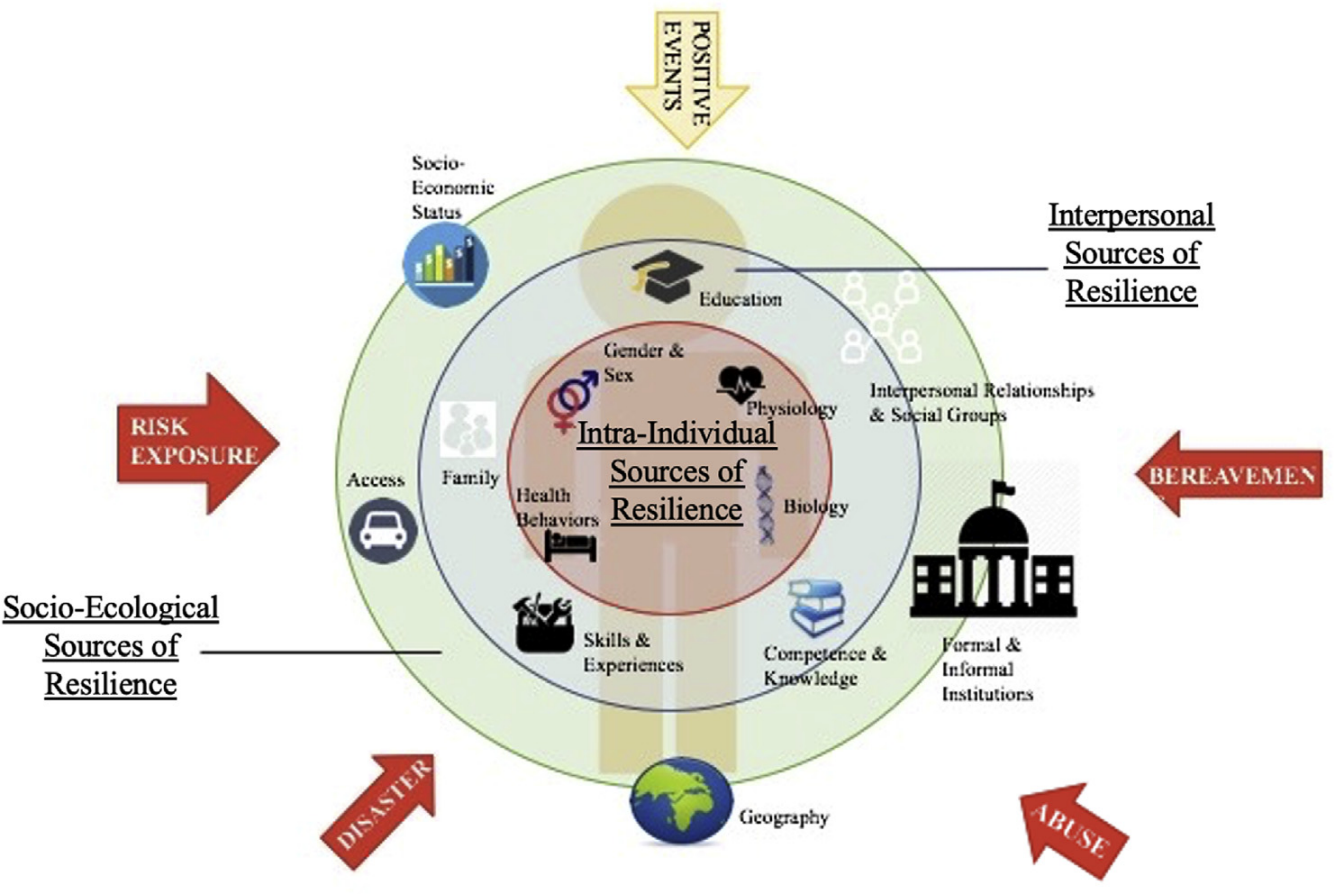
The original multi-system model of resilience (Liu et al., 2017).

To reflect the changing understanding of resilience as multidimensional (Bryant, 2015; Masten, 2015), the Multi-System Model of Resilience (MSMR) proposed the conceptualization of resilience as an evolving capacity that can be sourced from multiple areas or domains (Liu et al., 2017). The MSMR captures the factors that contribute to the process of resilience by identifying sources that influence and shape resilience (Figure 1). In response to the ongoing debates about what constitutes ‘resiliency’ – whether as traits, protective factors, psychological personality-correlates, and/or external social and community structures, the MSMR maps all of the above as *sources of resilience capacity* through three systems (Liu et al., 2017). All of these resources can be mobilized in the event of potential challenges or exposures to risks and adversities. In the absence of a stress or challenge, the systems are still able to delineate pathways to resilience, or “hidden resilience”, in order to maintain wellbeing (Ungar, 2006). For the purpose of this paper, resilience and resiliency will be used interchangeably in reference to the manifestations, conceptualizations, and operationalizations of resilience.

The MSMR was hypothesized to be comprised of three systems that act as the sources of resilience. Core resilience is conceptualized as the innermost system that includes health and health-related sources that are trait-like in nature and serve as a relatively stable foundation of resilience throughout life. This system has been renamed “Internal Resilience” to emphasize the sources as nested within the individual. The outermost system is the external resilience, consisting of socio-ecological sources of resilience. This system acts as structural determinants of resilience, including access to services, healthcare, and community-level social infrastructures that individuals are nested within. In between the internal and external is a system that reflects on an individual's orientation and response towards life and their external environment and circumstances. Serving to bridge the two systems of resilience, this layer is now termed “Coping & Pursuits” to highlight its dynamic and changing nature. Together, these hypothesized systems work to potentially identify conditions for which resiliency can manifest through diverse pathways (Liu et al., 2017).

## 2 Advancing the Multi-System Model of Resilience

We proposed that resilience can be defined as a reservoir of capacities and factors that enable adaptive functioning in various situations and conditions. A particular strength of the model is that it can be complementary to the various definitions of resilience already existing in the literature, whether posited as a trait-like disposition, a psychological personality correlate, or a functional state following the experience of trauma. To validate this hypothetical model, the current paper explores the empirical structure of the MSMR through a self-reported inventory along with existing measures of wellbeing and resilience.

### 2.1 Participants and procedures

Three samples of university students enrolled in introductory psychology courses through a university-wide online participation pool took part in this study after providing informed consent. Participants were diverse in their ethnic background, academic discipline, and grade point averages. Participants received course credit for study completion. The study obtained ethics approval from Ryerson University Research Ethics Board (REB 2017–230) in compliance with guidelines of the Canadian Psychological Association, and following the Declaration of Helsinki.

Sample One comprised of a total of 200 male and female students with a mean age of 20 (*SD* = 4.26). Given the large proportion of females in sample one (84%), we also ran the same statistical analyses after removing all the males, resulting in a total of 168 female students with a mean age of 20 (*SD* = 4.35) as Sample 1b. Sample Two is made up of 270 female university students, with a mean age of 20 (*SD =* 4.54). Finally, Sample Three is a smaller sample of 86 male-only university students, with a mean age of 22 (*SD =* 7.75).

### 2.2 Measures of resilience

In addition to the collection of demographic information, including age, sex, and ethnoracial backgrounds, participants completed various self-reported measures of resilience below using different approaches to operationalize resilience, including as protective factors, psychological domains, and individual difference variables (see Ahern et al., 2006 for review). Resilience measures used included:

1. *Multi-System Model of Resilience Inventory* (MSMR-I). The MSMR-I is a 27-item tool to measure resilience. Items were constructed to represent the three systems proposed by the MSMR, and the scale has been piloted for redundancy and comprehension. The items include coping skills, education, physical and mental health, socioeconomic status, and access to formal and informal support and institutions (Liu et al., 2017). Questions are scored on a 4-point Likert-type scale, with 0 corresponding to “not at all like me”, and 3 to “very much like me”. The internal consistency for MSMR-I was excellent in our samples, ranging from Cronbach's *α* = .90 to .91 overall, and .75 to .85 for the three systems within the MSMR (see Table S1).
2. *Baruth Protective Factor Inventory* (BPFI). The BPFI is an assessment of resilience through four protective factors: adaptable personality, supportive environment, presence of stressors, and compensating experiences (Baruth and Carroll, 2002). The BPFI consists of 16 items rated on a 5-point Likert-type scale. Although the BPFI has not been validated in larger samples, it is one of the only measures that examines protective factors (Smith-Osborne and Bolton, 2013). The internal consistency for BPFI for sample one was poor, Cronbach's *α* = .53. This measure was used with our original sample but was not used in subsequent samples due to poor psychometrics.
3. *Connor-Davidson Resilience Scale* (CD-RISC 25). The CD-RISC 25 contains 25 items scored on a 5-point Likert-type scale (Connor and Davidson, 2003). The CD-RISC assesses five factors, including: personal competence, high standards, and tenacity; trust in instinct, tolerance and strengths; positive acceptance of change and secure relationships; control; and spiritual influence. The CD-RISC has been validated in multiple populations, and has been particularly robust in health-care settings when assessing changes in response to treatment (Smith-Osborne and Bolton, 2013). The internal consistency for CD-RISC was acceptable, Cronbach's *α* = .86.
4. *The Resilience Scale* (RS-25). The RS is one of the most used measures of resilience, consisting of 25-items measuring factors of personal competence and acceptance of self and life (Wagnild and Young, 1993). The RS is scored on a 7-point Likert-type scale, and has been validated in individuals of all ages and backgrounds (Smith-Osborne and Bolton, 2013). The internal consistency for RS-25 was acceptable, Cronbach's *α* = .82.

### 2.3 Statistical analyses

Quantitative analyses were conducted using IBM's Statistical Package for the Social Sciences (SPSS) Software®, and the Psych and Lavaan packages in R. Exploratory analyses were conducted with sample one data, while confirmatory analyses were conducted with sample two and three data. All other psychometric analyses were conducted with data in all samples.

## 3 Findings of the Multi-System Model of Resilience

### 3.1 Exploring structures of the MSMR

**Figure 2:**
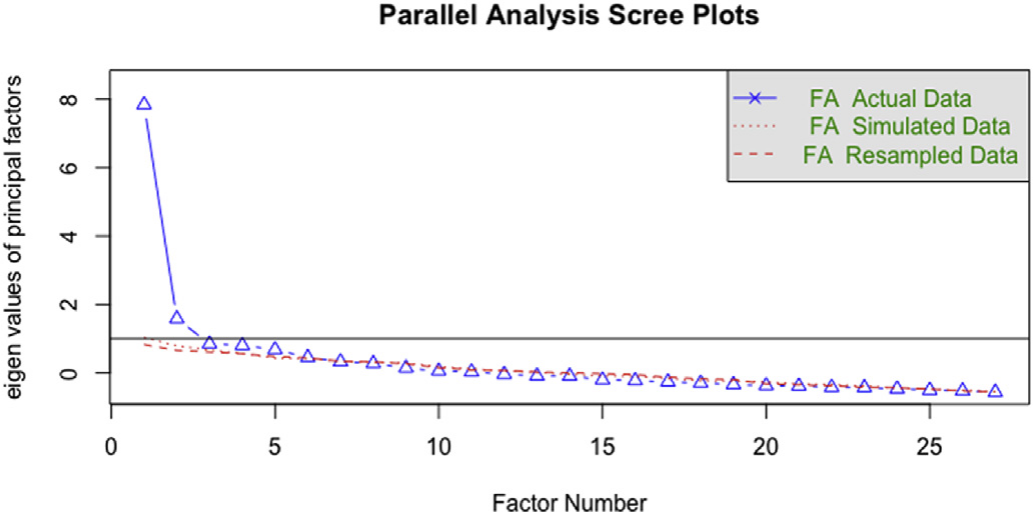
Parallel analysis of multi-system model of resilience.

A parallel analysis was conducted on Sample One to test the number of data-driven dimensions present in the items of MSMRI. Simulation outcomes of the parallel analyses suggested 2–5 factors based on eigen values of principle factors as illustrated in Figure 2. The number of suggested factors loosely correspond to the theorized three-systems model of MSMR.

To further examine the structure of the model, exploratory factor analysis with unweighted least squares (ULS) method and an oblimin rotation was performed on items of the MSMR-I. Model fit was examined using comparative fit index (CFI), Tucker-Lewis Index (TLI), root mean square error of approximation (RMSEA) and standardized root mean square residual (SRMR), the latter of which used the recommended cut-off of 0.08 (Marsh et al., 2004). Given the unequal distribution of males to females in Sample One, the first EFA was conducted on Sample 1b, and provided some support for a three-system structure of the overall model, CFI = 0.97, TLI = 0.97, SRMR = 0.09, RMSEA = 0.05, 90% CI = 0.04, 0.06. Confirmatory factor analysis was conducted with samples two and three to similar results (Table 1a). A review of these incremental fit indexes via CFI, TLI, SRMR, RMSEA suggested the three-systems model may be a good, but not great fit to the proposed conceptual model in all samples. Model fit statistics for Samples Two and Three are presented in Table 1a, while factor loadings for all three samples are presented in Table 1b.

**Table 1a:** Confirmatory model fit statistics.

	CFI	TLI	SRMR	RMSEA	90% CI
Sample Two	0.961	0.957	0.091	0.057	0.050, 0.065
Sample Three	0.996	0.996	0.121	0.014	0.000, 0.044

*Note.* CFI = comparative fix index, TLI = Tucker-Lewis Index, RMSEA = root mean square error of approximation; SRMR = standardized root mean square residual; CI = confidence interval.

**Table 1b:** CFA factor loadings for proposed systems.

Items	Loading Estimates		
	Sample 1b	Sample 2	Sample 3
Internal Resilience			
I eat a balanced diet	.685	.507	.698
I look after my body	.629	.478	.603
Item 3	.841	.706	.644
I struggle with my mental health	.762	.686	.449
I struggle with health problems	.492	.431	.367
Item 6	.507	.516	.438
I have problems with anger	.602	.457	.559
I find it hard to control my emotions	.553	.596	.428
Item 9	.541	.550	.451

Coping & Pursuits			
My life is meaningful	.455	.559	.538
I have a purpose in life	.513	.425	.606
Item 12	.355	.292	.295
I explore new ideas	.591	.684	.652
I seek new challenges	.685	.691	.735
Item 15	.581	.590	.634
I worry about the way I look	.698	.599	.988
I dwell on my past failures	.634	.586	.529
Item 18	.752	.566	.909

External Resilience			
I am constantly worried about money	.652	.856	.708
If an unexpected expense comes up, I worry about how I can pay for it	.898	.903	.762
Item 21	.757	.580	.668
I feel in control of my own life	.355	.393	.375
I feel that I belong	.379	323	.499
Item 24	.666	.654	.641
I know where to look for help if I need it	.651	.640	.497
Most of the services I need are accessible to me	.488	.535	.553
Item 27	.750	.704	.517

### 3.2 Model construct validity

Convergent validity was tested using Pearson's correlations. Data from Samples One and Two were used to explore the relationship of MSMR and its systems with existing measures of resilience. Table 2 displays the correlations among these measures. The MSMR and its systems (internal, coping & pursuits, external) significantly and positively correlated with all existing measures of resilience (*p* < .05). The strength of the correlation between MSMR and other measures of resilience ranged from weak to strong, suggesting variabilities in the relationships between measures.

**Table 2:** Pearson's Correlations between MSMR systems with measures of resilience.

	MSMR-Overall	MSMR Internal	MSMRCoping & Pursuits	MSMR External	CD-RISC	RS	BPFI
Sample One							
MSMR Overall	--	--	--	--	--	--	--
MSMR Internal	.858∗∗	--	--	--	--	--	--
MSMR Coping & Pursuits	.915∗∗	.661∗∗	--	--	--	--	--
MSMR External	.901∗∗	.649∗∗	.768∗∗	--	--	--	--
CD-RISC	.725∗∗	.576∗∗	.761∗∗	.589∗∗	--	--	--
RS	.636∗∗	.543∗∗	.661∗∗	.486∗∗	.793∗∗	--	--
BPFI	.314∗∗	.231∗∗	.330∗∗	276∗∗	.550∗∗	.531∗∗	--

Sample Two							
MSMR Overall	--	--	--	--	--	--	--
MSMR Internal	.853∗∗	--	--	--	--	--	--
MSMR Coping & Pursuits	.912∗∗	.689∗∗	--	--	--	--	--
MSMR External	.844∗∗	.569∗∗	.680∗∗	--	--	--	--
CD-RISC	.755∗∗	.555∗∗	.775∗∗	.643∗∗	--	--	--
RS	.652∗∗	.522∗∗	.642∗∗	.532∗∗	.781∗∗	--	--

*Note*. MSMR = Multi-System Model of Resilience; CD-RISC = Connor-Davidson Resilience Scale 25; RS = Resilience Scale; BPFI = Baruth Protective Factor Inventory.

∗∗*p* < .001.

To further examine the relationship of MSMR systems as related to other constructs of resilience, we conducted CFAs using MSMR Internal, Coping & Pursuit, and External, along with other measures of resilience in both samples. In Sample One, a three-factor model of resilience explained 87.47% of the total variance. An examination of the factor loadings identified MSMR External Resilience to be a unique factor that does not share any cross-loadings with other resilience measures (see Table 3). The second factor appear to be driven by BPFI, while MSMR Internal Resilience, MSMR Coping & Pursuits, CD-RISC, and RS loaded onto the third factor.

**Table 3:** Construct validity of MSMR through CFA loadings.

Measures	Factor		
	1	2	3
Sample One – Three-Factor Model			
MSMR-IR	.628	.177	**.677**
MSMR-CP	.749	.261	**.841**
MSMR-ER	**.993**	.159	.664
CD-RISC	.522	.475	**.904**
RS	.429	.499	**.863**
BPFI	.144	**.995**	.525

Sample One – Two-Factor Model			
MSMR-IR	--	**.999**	.663
MSMR-CP	--	.644	**.806**
CD-RISC	--	.568	**.948**
RS	--	.545	**.836**

Sample Two – Three-Factor Model			
MSMR-IR	.054	**.749**	.642
MSMR-CP	.197	.267	**.842**
MSMR-ER	**.650**	.082	.727
CD-RISC	-.011	-.105	**.948**
RS	-.272	-.105	**.912**

Sample Two – Two-Factor Model			
MSMR-IR	--	**.771**	.582
MSMR-CP	--	.413	**.811**
CD-RISC	--	.017	**.952**
RS	--	-.122	**.931**

*Note*. MSMR = Multi-System Model of Resilience; IR = Internal Resilience; CP = Coping Pursuits; ER = External Resilience; CD-RISC = Connor-Davidson Resilience Scale 25; BPFI = Baruth Protective Factor Inventory; RS = Resilience Scale. Factor loadings are bolded.

As BPFI had poor psychometrics compared to other measures of resilience, CFA was conducted again with only MSMR Internal, Coping & Pursuits, CD-RISC, and RS (as MSMR External had already loaded onto its own factor). This resulted in a two-factor solution that accounted for 87.87% of the variance, with MSMR Internal loading onto a unique factor, while MSMR Coping & Pursuits, CD-RISC, and RS loading onto another factor. CFAs with Sample Two data (without BPFI) resulted in similar factor loadings, accounting for a total of 91.52% and 83.28% of variance with a 3-factor model and a 2-factor model respectively. Factor loadings for both samples are presented in Table 3.

## 4 General discussion

Findings of the current study provide some empirical support for the theoretical model of MSMR and the proposed three-systems structure. Generally, model fit statistics indicate that a three-factor model can be a moderately-good fit for the data, with areas for improvement. Importantly, the three-factor structure appears relatively stable and consistent within the samples tested, as well as provide an overarching framework that complements existing measures of resilience. These findings, taken together with the strong theoretical rationale for the three-systems structure, highlight the importance of tapping into the multidimensionality of resilience, and underscores the importance for future investigations and refinements in model developments.

The exploration of the relationship among the MSMR systems with existing measures of resilience found that the MSMRI was significantly positively correlated with all other measures of resilience. In addition, subsequent analyses found the three systems within MSMR to share varying degrees of overlap with existing measures and concepts of resilience, with some distinctions that served to highlight the advantage of MSMR as a novel conceptualization of resilience.

### 4.1 Understanding systems of resilience

**Figure 3:**
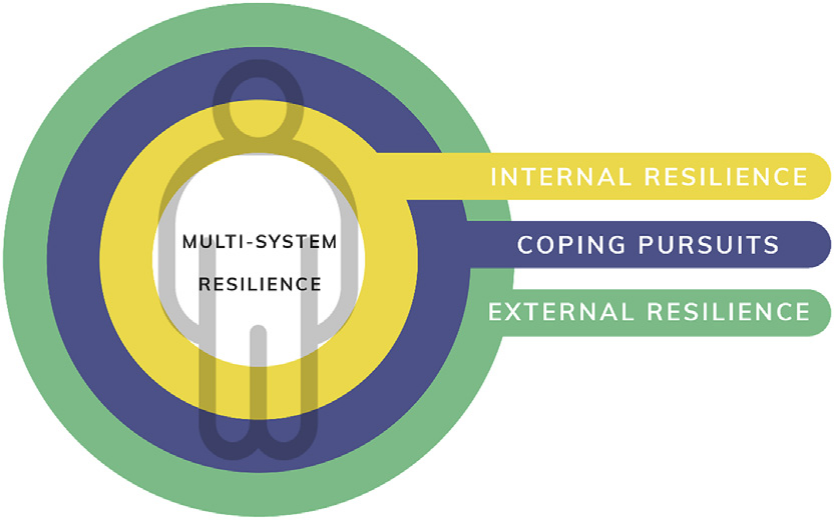
Updated multi-system model of resilience.

Based on the current analysis, systems within the MSMR are comprised of: 1) Internal Resilience, sources of resilience within the individual, including health and health-related factors that promote wellbeing; 2) Coping & Pursuits, coping-related skills, knowledge, and goals that allow the individual to respond to challenges and needs; and 3) External Resilience, socio-ecological factors that promote resilience, such as access to healthcare and support services (see Figure 3).

Internal Resilience encompasses the sources of resilience that are inherently nested within the individual, such as psychological and physical health. Within this system, health indicators that act as foundational sources of resilience capacity are captured. Individual-level determinants of health, such as health functioning, mindsets, attitudes, and perceptions of one's own health continues to evolve and shape subsequent health-related behaviours (Short and Mollborn, 2015). Psychological factors that shape health behaviours, and/or reinforce health outcomes thus fall within this system. In this regard, IR shares some overlapping properties with other measures of psychological resilience, such as the CD-RISC and RS. Extending beyond self-report indicators of IR, there has been increasing research focused on uncovering the genetic and neurobiological determinants of individual health and behaviours as they relate to manifestations of resilience (e.g., Basner et al., 2013; Cong et al., 2017; Osório et al., 2017). Despite these advancements, few conceptualizations of resilience incorporate these findings as determinants of individual resilience capacity (Osório et al., 2017). Instead, understanding of resilience often are limited to psychological while failing to capture the feedback loop and interrelated dynamics among an individual's biophysiological health such as genetic and neurochemical make-up, processes or outcomes that shapes health-related cognitions, and subsequent coping in response to challenges and events (Osório et al., 2017).

Meanwhile, Coping and Pursuits, at the conceptual level, shares the most similarities with existing measures and conceptualizations of resilience. Indeed MSMRI-CP's emphasis on psychological and interpersonal factors that shape individual variations in coping preferences, styles, and cognitions are captured empirically as the items within this system shared the most variance with alternative measures of resilience, such as CD-RISC and RS. Although coping factors such as optimism, creativity, and meaning-making are key determinants in shaping behavioural responses following trauma, they should not be considered in a vacuum independent of individual health, or larger socio-structural determinants that provide the backdrop to contextualize integrative resilience (Masten, 2015). Indeed, both of the systems (internal and external) cyclically inform coping behaviours, styles, and patterns through individual experience and exposures throughout the lifespan (Bonanno et al., 2015). For example, prolonged exposure to stress may result or be resultant of individual coping styles, which may also be informed by maladaptive health behaviours and further limited by lack of available healthcare or supportive services.

Indeed, the External Resilience system positions the individual within a larger socio-structural system, and highlights the structural determinants of health and wellbeing as important sources of resilience. The examination of determinants of resilience at levels beyond the individual have been identified as a key missing link and an important area of future focus in resilience research (Kimhi and Eshel, 2015; Masten, 2016; McNally, 2015). Based on findings from the current paper, ER appears to be a relatively unique factor with less shared variance to existing measures. While none of the systems are intended to be regarded as a self-contained and insulated from the influence of others, their theoretical organization within the MSMR may help guide future research into the distinctions in how they may be sourced, the relative stability (or instability) of the systems under different contexts, as well as the interplay of factors that may shape them over time.

Within the MSMR, resilience is not contingent on the presence of adversity or stressors. Adverse events act as triggers or exacerbators, undermining or depleting sources of internal and external resilience, taxing our abilities to cope adaptively. Individual resilience is determined by the ability to pivot/match needs based on one's resources. For example, individuals who have less internal resources (e.g., chronic illness resulting in poor physical health) may be able to draw on their ample external resources (e.g., financial security and social support) to compensate when challenged. Others rich in internal resilience resources (e.g., strong self-regulation) may also be able to maintain high resilience despite deficits in external resources (e.g., living in poverty). Finally, individuals can meet their varying needs and challenges with appropriate coping skills and strategies to compensate for any deficiencies in internal or external resilience. When either internal or external resilience resources are deficient and the coping pursuits can do little to make up for these deficiencies, an individual may experience high vulnerability and low resilience. Although they may still be functioning, the accessible reservoir of resilience is low. Once this person experiences a challenge, there is little reserve to draw from, making them susceptible to a range of negative outcomes, such as depression or anxiety.

#### 4.2 Challenges and future considerations

The goal of the MSMR was to propose a framework for conceptualizing resilience as a multidimensional capacity sourced from three systems. The MSMR intends to complement existing approaches of measuring and understanding resilience, whether as an individual trait, a personality correlate of coping, or as recovery or a desired outcome of positive trajectory following exposure to adversity (Liu et al., 2017). A persistent challenge in the resilience literature continues to be the variability in conceptualizations that fail to explain how individuals facing the same adversity may experience differences in outcomes, and how the same individual facing multiple adversities will cope differently to each adversity encountered. Within these contexts, the MSMR model provides some insights into how the systems may work together to facilitate various trajectories in response to challenges, and offers some explanations to support how different recoveries may be achieved following exposure to various adversities or stressors based on the availability of and mobilization of resilience resources. It may be that within each of the systems, there may be alternative or nested structures that guide the utilization of resilience sources. The model fit statistics could be further improved by considering nesting factors and hierarchical models. Future investigations and further advancements are needed in order to delineate exactly how these systems of resilience interplay to meet varying needs.

Although findings presented in the paper lend some support for the theoretical model, a number of challenges and limitations were encountered. A persistent challenge during this research process was the construction of a quantitative tool which taps into the model and appropriately represents its distinct systems. Authors Mendenhall and Kim (2019) noted difficulties in adapting an existing resilience scale to be inclusive and representative for research examining socio-cultural resilience in the community. While a particular advantage of the MSMR is the inclusion of external resilience as a system, we also recognize the limitations in quantifying a model entirely through self-report. However, the use of self-report is an important first step in quantifying a theoretical model. The measure will need to undergo refinements and adaptations along with additional expansions of quantitative measurements of resilience, such as genomics and neurological mechanisms of cellular resilience (Choi et al., 2019; Riordan and Nadeau, 2017), mapping of community social structures (Ungar, 2018), and tiered structures of assessing risks and resilience (Linkov et al., 2018) to supplement the self-report inventory.

Interpretation of findings from the current paper should also be made in consideration of study limitations. First, the sampled populations were predominantly female students. Past research has documented variations in the types and levels of resilience observed across different genders (e.g., Boardman et al., 2008), age (e.g., Hayman et al., 2017), and culture (e.g., Ungar, 2008). While results did not show large variations between male and female samples, further research should be conducted to explore the structures of resilience in diverse populations and explore potential variations in the modelling of the MSMR. Further, while student populations have often been used in research (e.g., Fung, 2020), their exposures to stressors and challenges, and their risks for mental illness and other negative health outcomes may not be representative or generalizable to other population groups (Eisenberg et al., 2007). Research with MSMR should explore its factor substructure and examine characteristics that may lead to differences in resilience systems in other populations, including diverse groups based on gender, age, risk exposures, and cultural backgrounds.

Advancements in model and measure developments could also consider sensitive time periods in lifespan trajectories and their implications for overall as well as system-specific sources of resilience, such as considering neurobiological sensitivity in early development, and effects of chronic stress exposures for later life resilience (Linkov et al., 2020). More work is needed to fit other types of data into the existing model, including the addition of experience sampling, biophysiological measures, and structural-level data on the availability of various social and healthcare services. Finally, applications of the MSMR could also examine its utility across various situations and contexts, such as assessment of risks, safety management, and adoptions during pandemics and other events.

### 4.3 Conclusion

Resilience, as defined within the MSMR, represents an evolving capacity to respond to challenges or traumas over time, with the supposition that resilience may be sourced from a combination of factors depending on varying needs, situational demands, and availability of resources. Findings from the current paper are consistent with the proposed scope of the conceptual model and highlight the importance of examining contributors to resilience capacity through processes that extend beyond individual-level coping. The systems within MSMR work together to delineate differences in pathways to resilience, with capacity for resilience evolving from one context to another. The conceptualization of MSMR as a reservoir of capacity that enables functioning on a continuum from vulnerability to resilience is particularly adaptable and complementary to existing understandings of resilience, as well as offer insights as to why different individuals facing the same adversity may experience different outcomes, and why the same individual may demonstrate different resilience levels from time to time in response to changes encountered throughout his or her lifetime.

## Declarations

### Author contribution statement

J. J. W. Liu: Conceived and designed the experiments; Performed the experiments; Analyzed and interpreted the data; Contributed reagents, materials, analysis tools or data; Wrote the paper.

M. Reed, K.P. Fung: Conceived and designed the experiments; Contributed reagents, materials, analysis tools or data; Wrote the paper.

### Funding statement

This work was supported by the Partnership for Change: The 10.13039/100012395Royal Bank of Canada Immigrant, Diversity and Inclusion Project Grant at 10.13039/100007891Ryerson University, and the 10.13039/501100000108Psychology Foundation of Canada/10.13039/501100003284Canadian Psychological Association Student Research Grant.

### Competing interest statement

The authors declare no conflict of interest.

### Additional information

No additional information is available for this paper.

## USE OF MSMR-I

The MSMR-I is available by request. Please contact the corresponding author for use and permissions.
