# Supplementary material for: Advancements to the Multi-System Model of Resilience: updates from empirical evidence
Source: Heliyon. 2020 Sep 14;6(9):e04831. doi: 10.1016/j.heliyon.2020.e04831 (PMC7492804; doi:10.1016/j.heliyon.2020.e04831)
Supplement: Table S1 [file mmc1.docx]

Table S1. *Descriptive and psychometric properties of the MSMR-I across studies*

|  | **Mean** | **Std. Dev.** | **Min** | **Max** | **Variance** | **Skewness** | **Kurtosis** | **Cronbach’s *α*** | **Mean item inter-correlation** |
| --- | --- | --- | --- | --- | --- | --- | --- | --- | --- |
| *MSMR-I Total* |  |  |  |  |  |  |  |  |  |
| Sample 1 (males and females) | 49.09 | 14.02 | 11 | 79 | 196.44 | -.278 | -.329 | .91 | .27 |
| Sample 1b (females) | 48.17 | 14.18 | 11 | 79 | 201.05 | -.246 | -.355 | .91 | .27 |
| Sample 2 (females) | 45.87 | 14.06 | 8 | 78 | 197.63 | -.164 | -.237 | .91 | .28 |
| Sample 3 (males) | 50.71 | 13.12 | 20 | 81 | 172.14 | -.055 | -.456 | .90 | .26 |
| *Internal Resilience* |  |  |  |  |  |  |  |  |  |
| Sample 1 (males and females) | 16.78 | 5.06 | 2 | 27 | 25.56 | -.522 | -.141 | .78 | .27 |
| Sample 1b (females) | 16.47 | 5.18 | 2 | 27 | 26.81 | -.484 | -.218 | .79 | .29 |
| Sample 2 (females) | 15.88 | 4.95 | 3 | 27 | 24.55 | -.242 | -.199 | .79 | .29 |
| Sample 3 (males) | 17.57 | 5.01 | 3 | 27 | 25.10 | -.477 | -.120 | .81 | .32 |
| *Coping Pursuits* |  |  |  |  |  |  |  |  |  |
| Sample 1 (males and females) | 15.36 | 5.60 | 2 | 27 | 31.33 | -.062 | -.618 | .82 | .34 |
| Sample 1b (females) | 15.04 | 5.67 | 2 | 27 | 32.17 | -.026 | -.656 | .83 | .36 |
| Sample 2 (females) | 13.97 | 5.76 | 0 | 27 | 33.13 | -.049 | -.519 | .85 | .38 |
| Sample 3 (males) | 15.87 | 4.93 | 4 | 27 | 24.28 | .013 | -.263 | .75 | .26 |
| *External Resilience* |  |  |  |  |  |  |  |  |  |
| Sample 1 (males and females) | 16.96 | 5.05 | 4 | 27 | 25.53 | -.218 | -.385 | .77 | .27 |
| Sample 1b (females) | 16.67 | 5.11 | 2 | 27 | 26.09 | -.200 | -.400 | .78 | .28 |
| Sample 2 (females) | 16.02 | 5.27 | 0 | 27 | 27.80 | -.310 | .013 | .79 | .30 |
| Sample 3 (males) | 17.14 | 4.83 | 7 | 27 | 23.34 | .168 | -.656 | .77 | .28 |

*Note. MSMR-I =* Multi-System Model of Resilience – Inventory; *α* ***=*** alpha.
